# Identification of multiplicatively acting modulatory mutational signatures in cancer

**DOI:** 10.1186/s12859-022-05060-8

**Published:** 2022-12-06

**Authors:** Dovydas Kičiatovas, Qingli Guo, Miika Kailas, Henri Pesonen, Jukka Corander, Samuel Kaski, Esa Pitkänen, Ville Mustonen

**Affiliations:** 1 Institute for Molecular Medicine Finland (FIMM), HiLIFE, University of Helsinki, 00014 Helsinki, Finland; 2 Department of Computer Science, University of Helsinki, 00014 Helsinki, Finland; 3 Organismal and Evolutionary Biology Research Programme, University of Helsinki, 00014 Helsinki, Finland; 4 Helsinki Institute for Information Technology, University of Helsinki, 00014 Helsinki, Finland; 5 Institute of Biotechnology, University of Helsinki, 00014 Helsinki, Finland; 6 Applied Tumor Genomics Research Program, Research Programs Unit, University of Helsinki, 00014 Helsinki, Finland; 7 Institute of Basic Medical Sciences, University of Oslo, 0317 Oslo, Norway; 8 Parasites and Microbes, Wellcome Sanger Institute, Hinxton, CB10 1SA UK; 9 Department of Mathematics and Statistics, University of Jyväskylä, 40014 Jyväskylä, Finland; 10 Department of Computer Science, Aalto University, 00076 Aalto, Finland; 11 Department of Computer Science, University of Manchester, Manchester, M13 9PL UK

**Keywords:** Mutational signatures, Modulatory processes, Cancer

## Abstract

**Background:**

A deep understanding of carcinogenesis at the DNA level underpins many advances in cancer prevention and treatment. Mutational signatures provide a breakthrough conceptualisation, as well as an analysis framework, that can be used to build such understanding. They capture somatic mutation patterns and at best identify their causes. Most studies in this context have focused on an inherently additive analysis, e.g. by non-negative matrix factorization, where the mutations within a cancer sample are explained by a linear combination of independent mutational signatures. However, other recent studies show that the mutational signatures exhibit non-additive interactions.

**Results:**

We carefully analysed such additive model fits from the PCAWG study cataloguing mutational signatures as well as their activities across thousands of cancers. Our analysis identified systematic and non-random structure of residuals that is left unexplained by the additive model. We used hierarchical clustering to identify cancer subsets with similar residual profiles to show that both systematic mutation count overestimation and underestimation take place. We propose an extension to the additive mutational signature model—multiplicatively acting modulatory processes—and develop a maximum-likelihood framework to identify such modulatory mutational signatures. The augmented model is expressive enough to almost fully remove the observed systematic residual patterns.

**Conclusion:**

We suggest the modulatory processes biologically relate to sample specific DNA repair propensities with cancer or tissue type specific profiles. Overall, our results identify an interesting direction where to expand signature analysis.

**Supplementary Information:**

The online version contains supplementary material available at 10.1186/s12859-022-05060-8.

## Background

After the first whole genome mutational catalogues of cancer samples were described a decade ago, a breakthrough development has been the conceptualization and identification of mutational signatures [1–3]. A mutational signature is the imprint left on cancer genome by a mutagenic process active over the course of cancer development [2, 3]. For instance, UV-light generates a distinct pattern of somatic mutations in melanoma genomes (characterised by C:G>T:A at specific nucleotide contexts). There has been substantial progress in categorizing these mutation patterns by their endogenous and exogenous mutagenic causes through analysis of large-scale cancer sequencing datasets [4] and observing effects of specific mutagens in a controlled environment [5]. The dynamic change of active mutational signatures during tumour evolution holds great potential to guide therapeutic strategies in personalised medicine [6–11]. For example, a cell population showing activity of a signature indicating a defect in DNA double stranded break repair would potentially respond to either platinum therapy or PARP inhibitors [8, 12].

The cornerstone of mathematical analysis of mutational signatures is the non-negative matrix factorization (NMF) [13, 14]. Using NMF-based framework, a state-of-the-art single-base substitution (SBS) mutational signature catalogue was recently derived from pan-cancer analysis of 2,780 whole genomes (PCAWG) across 37 tumour types (version 3, released May 2019 as part of COSMIC v89) [4, 15]. These signatures can explain the observed somatic mutation counts with an average reconstruction accuracy (cosine similarity) of 0.97 in PCAWG data, using just four mutational signatures per sample on average [4]. In this framework, the joint mutational catalogue of a sample is explained as a sum over mutational signatures each multiplied by their respective activities. Therefore, the NMF paradigm-based mutational signature model is inherently additive. This means that the NMF-based model cannot distinguish between DNA damage and DNA repair processes that can act in a manner of removing mutations in cancer cell DNA. As mutational catalogues are a combination of DNA damage and repair processes [16], the representation of known mutational signatures may already have been influenced by DNA repair mechanisms. Furthermore, these repair processes may be selectively active in different cancer genomes, and have cancer or tissue type specific profiles. Repair processes would thus be ideally treated independently from DNA damage processes. Consequently, there is a need for extending the additive model to account for repair processes, while keeping the basis of the successful, biologically interpretable, NMF mutational signature model intact. To this end, we propose a multiplicative adjustment, i.e. rescaling the net outcome of additive mutation signatures by multiplying the profile with a modulatory mutational signature that can be iteratively learned from the observed mutation data using the log-likelihood maximization algorithm. We suggest that such modulatory processes biologically relate to cancer sample specific DNA repair propensities with tissue or cancer type specific profiles.

Firstly, we investigate to what extent the 96-channel mutational counts across the PCAWG-data are explained by the known mutational signatures by carefully examining the residuals, i.e., the differences between the observed and model-fitted mutation counts. To achieve this, we contrast the observed somatic mutation counts in the PCAWG-data against the reconstructed mutation counts by the NMF model [4]. Our results reveal systematic structure in the residuals over the 96 mutation types despite the high quality of the additive NMF model fits. We next focus on cancer samples that cluster in their residual profile and develop a probabilistic model to explain the residuals, i.e., cancer-specific multiplicatively acting modulatory mutational signatures. We then infer such modulatory signatures and their activities from somatic mutation data, applying it across the tumour types in PCAWG-data. We show that the aforementioned non-random structured residuals are almost fully removed by including a multiplicative process to extend the additive model.

## Results

### Residual analysis of the NMF model reveal unexplained structure

**Fig. 1:**
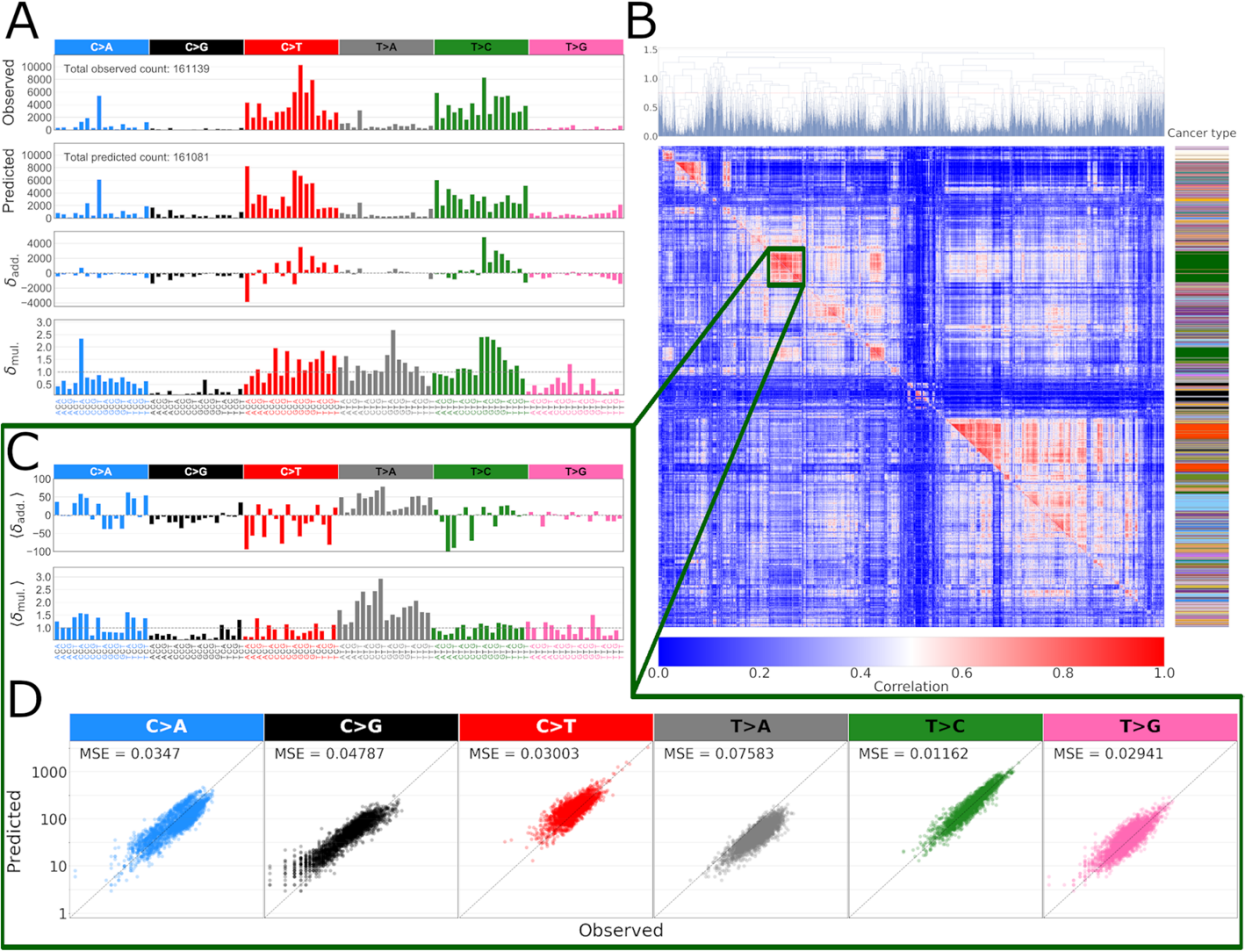
**A** Observed and predicted mutation counts (first and second panels), additive residual $X - {\widetilde{X}}$ (third panel) and multiplicative residual $X/{\widetilde{X}}$ (fourth panel), where *X* is observed and ${\widetilde{X}}$ is NMF model fitted mutation counts of one example sample (colorectal adenocarcinoma (ColoRect-AdenoCA) PCAWG sample SP16934). Bars are colored according to their base SBS type. **B** Multiplicative and additive residual correlation clustering. The upper triangle represents the multiplicative residual correlations, while the lower triangle represents the additive residual (the lower triangle correlation values are ordered according to the ordering of the upper triangle). Correlation scale is limited to the range of [0, 1]. The color-bar on the right show the cancer type (refer to Additional file 1: Fig. S1C for legend). **C** Mean residual profiles of the zoomed in block consisting mostly of Liver-HCC samples (see text). **D** A scatter plot of the mutation counts vs. NMF model predicted counts for this Liver-HCC cluster shows a systematic overestimation of C>G and underestimation of T>A mutations. These systematic biases are also evident from the mean multiplicative residual profile of panel **C**

We evaluated two residuals, an additive and a multiplicative one,

1 $$\begin{aligned} \delta ^k_{\mathrm {add.},j} = & {} X^k_j-\widetilde{X^k_j},\nonumber \\ \delta ^k_{\mathrm {mul.},j} = & {} X^k_j/\widetilde{X^k_j}, \end{aligned}$$

between the observed mutation data $X_j^k$ and the NMF model fits $\widetilde{X_j^k}$ from [4], for each sample *k* and feature *j*. This resulted in a 96-feature residual profile vector for each cancer sample (Fig. 1A).

To investigate the structure of both types of residuals we applied hierarchical clustering, using correlation as a similarity metric, (see “Hierarchical clustering” in “Methods” section) on the sample-wise residues of both types in the PCAWG-data. The clustering results revealed systematic residual structure (Fig. 1B), much of it shared between the two types of residues. As a control, we considered a scenario where the model would fully explain the observed mutation count data and the residues would consequently reflect only genuine stochasticity of the underlying mutational processes as given by the noise associated with the Poisson process [17]. To this extent, we re-sampled observed mutation counts vectors from the PCAWG data using the counts as Poisson rates. As expected, computing residuals between such a “perfect” model and the input data shows almost no residual structure upon clustering (Additional file 1: Fig. S2).

### Cluster analysis connects residuals structure with cancer type

Visual inspection of samples clustered by residuals Fig. 1B indicated clusters to contain same tumor types (201 clusters in total, see “Hierarchical clustering” in "Methods" section). We further performed a dimension reduction by uniform manifold approximation (UMAP [18]) of multiplicative residuals which also showed considerable clustering by cancer type Additional file 1: Fig. S1).

Next, we examined several individual clusters, calculating cluster specific average residual and mutation count profiles and inspecting mutation counts with respect to each mutation type. The first cluster we examined consists of mostly liver hepatocellular carcinomas (henceforth Liver-HCC cluster) (n = 159 out of 163 total) (Fig. 1C,D). In this cluster, both systematic underestimation and overestimation of the NMF model predicted mutation counts are visible in C>G and T>A trinucleotide contexts, respectively. The fact that the predicted mutation counts are displaced compared to the diagonal in these mutation contexts, resulting in hundreds (mean of 281 mutations per sample in C>G and 642 in T>A) of incorrectly predicted mutations, suggests that this deviation is inconsistent with random unbiased source, but rather a shortcoming of either the additive model itself or the mutational signature set.

To check to what extent the clusters tend to form cancer type specific groups, we ranked the clusters by their entropy as evaluated by cancer type labels. Clusters consisting of only one type of label would have zero entropy, whereas higher entropy values signify more varied label composition in the clusters. Several zero-entropy clusters, indicating a single cancer type, are formed by skin melanoma samples—(n = 18), (n = 17), (n = 14) and (n = 10)—and are characterized by distinctively high mutation numbers. However, despite these samples having similar overall mutation spectra, their residual profiles are quite different, possibly caused by different active mutational signature assignment based on associated clinical data or different mutagenic processes and hypermutability. The aforementioned Liver-HCC cluster is the lowest-entropy cluster (S = 0.141) aside from zero-entropy ones above. All clusters had a specific residual pattern suggesting a systematic, i.e. non-random, mutation count residue distribution. We used the same clustering methodology to analyse the alternative model fit of Bayesian-NMF based method *SignatureAnalyzer* results. Fewer cancer type-specific clusters are formed and they have lower residue correlations. This is expected given that *SignatureAnalyzer* model fit allows many more signatures to be active per sample (median is 15, compared to 4 in the *SigProfiler* fit). Nevertheless, the fit is, again, not free of systematic structure of the residues (Additional file 1: Fig. S5).

### Extending the additive NMF model to encompass multiplicative modulatory mutational signatures

The evident non-randomness of the mutation count residues suggests that the additive NMF model does not fully explain the observed mutations. If the number of mutations were just underestimated by the model, additional mutational signatures could be a solution. However, we also observe systematic mutation count overestimation. It follows that either the entire mutational signature set must be edited and refined, perhaps to separate DNA damage and DNA repair effects [16], or the additive model modified to correct for both underestimation and overestimation of mutations counts.

Here we extend the additive mutational signature model to include a multiplicative term, allowing for the correction of both overestimation and underestimation of the predicted mutation numbers. Biological justification for the modification is to model DNA repair processes that may have sample specific propensities, as well as cancer or tissue type specific profiles. For a *j*-th mutation type of the *k*-th sample, we add a multiplicative term $(1 + c^k r_j)$, where $r_j$ is the *j*-th multiplier of a global modulatory process and $c^k$ is the activity of that process in *k*-th sample. Then the observed counts are distributed as,

2 $$\begin{aligned} X^k_j \sim \textrm{Pois} ((1 + c^k r_j){\widetilde{X}}^k_j), \end{aligned}$$

where ${\widetilde{X}}^k_j = \sum _{i = 1}^{N_{\textrm{sig}}} a_i^k\mu _j^i$ is the standard additive part where each mutational signature, $\mu ^i$, contributes by its sample specific activity, $a_i^k$. The model allows for the modulatory process *r* to be inactive in any given sample by setting $c^k = 0$, returning then the additive model, ${\widetilde{X}}^k_j$, which is equivalent to the NMF model when Kullback-Leibler matrix norm is chosen [17].

### Modulatory process inference

#### Simulated data benchmark

**Fig. 2:**
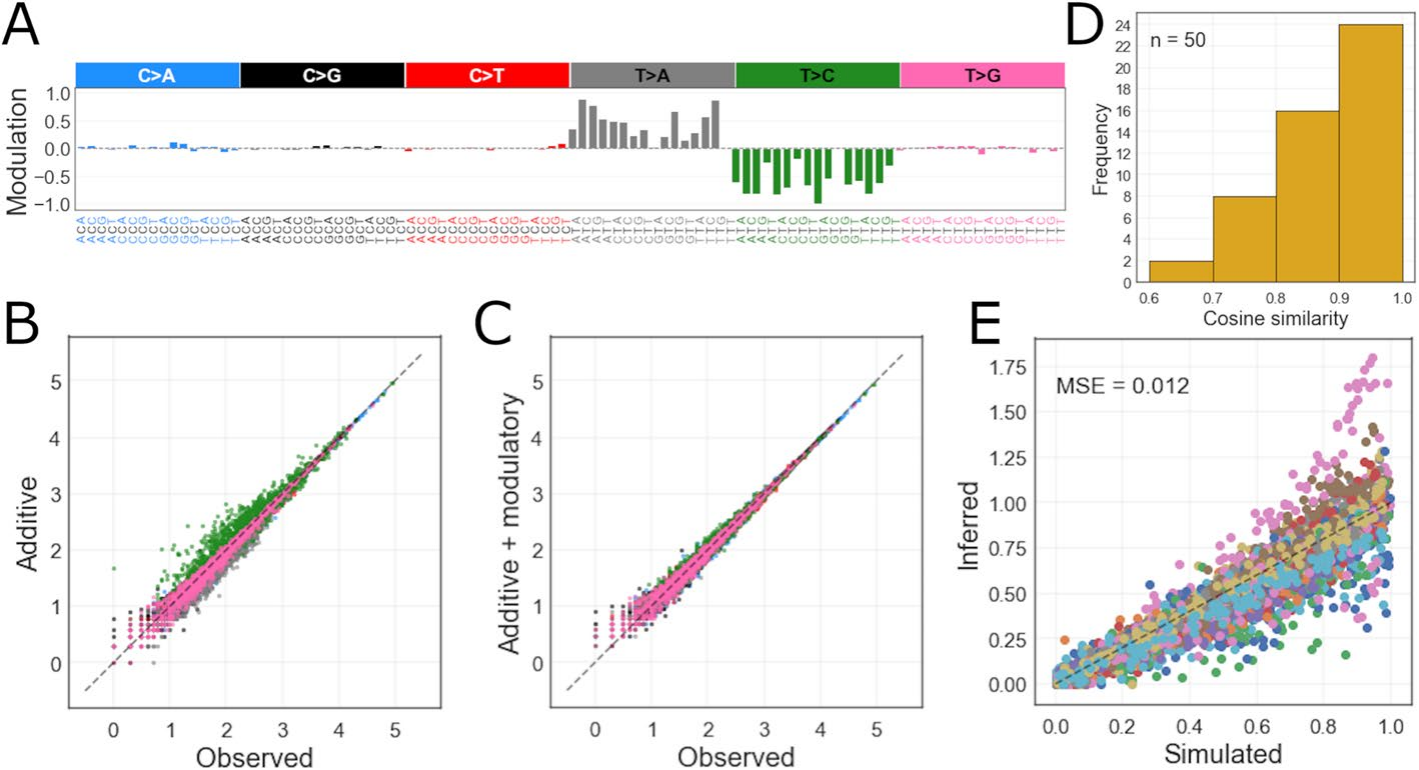
**A** An example of a simulated modulatory process: T>C mutation features are modulated downwards and T>A mutations are modulated upwards. **B** Simulated versus predicted mutation counts by additive model in one dataset (affected by the example modulatory signature). **C** Simulated versus predicted-modulated with the modulatory process mutation counts in the example dataset (corrected with the inferred modulatory process). **D** The cosine similarity of simulated and inferred modulatory signatures. **E** Simulated versus inferred modulatory process activity values for all 50 datasets (each dataset is colored differently)

We first performed maximum likelihood inference of simulated modulatory processes (Fig. 2A) and their sample specific activities. We generated 50 simulated modulatory process mutational signatures which were used to modulate the standard sample specific additive signatures model. This product was then used to draw a set of 100 cancer samples (each had their cancer specific additive signatures and their activities) for each modulatory process (see “Data simulation” in "Methods" section). In the inference, we took the additive signatures $\mu$ as given. The maximum likelihood method inferred the signature activities *a* as well as the data set specific modulatory signatures *r* with a mean cosine similarity of 0.86 (Fig. 2D), together with their activities *c* with a total mean squared error (MSE) of 0.012 (Fig. 2E). Figure 2B depicts a clear systematic discrepancy between the observed counts and additive model fits when the simulated data contains a modulatory process. The systematic errors mirror the underlying modulatory process (Fig. 2A), are closely reminiscent to the Liver-HCC cluster samples shown in Fig. 1C, and provide intuition on how would such effects manifest in the PCAWG data and the corresponding fits. In contrast, the extended model as given by Eq. 2 can fit the sample mutation counts without systematic errors (Fig.  2C).

Additionally, we compared the true mutational signature activities, used to generate the synthetic mutation catalogues, to the activities inferred under both models—additive (no modulatory process active) and extended (incorporating the inferred modulatory process). The per-sample (additive) activity vectors are essentially the same regardless of the model (mean cosine similarity 0.97 when comparing additive vs. extended model inferred activities), suggesting that signature re-fitting is not sufficient in explaining the variation induced by multiplicative interactions.

#### Inclusion of modulatory processes greatly improves the model fits

**Fig. 3:**
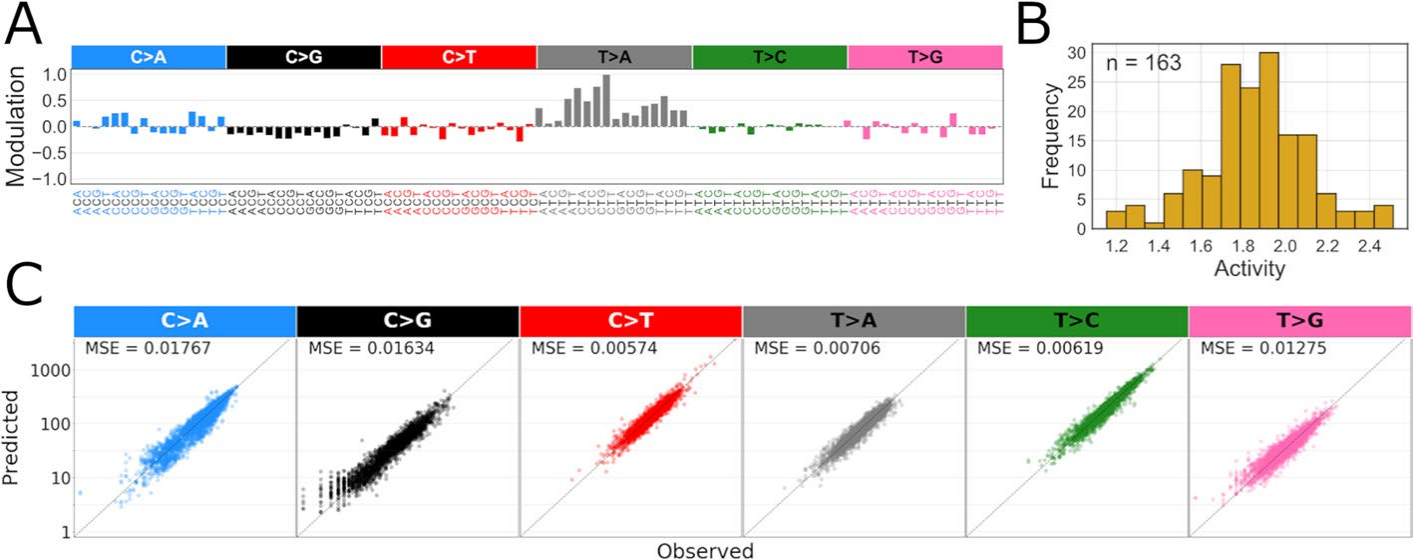
**A** The modulatory process *r* inferred for samples in Liver-HCC dominated cluster of PCAWG data shown in Fig. 1. **B** The corresponding modulatory signature activity histogram. **C** SBS base mutation type-specific scatter plots of observed vs. predicted (additive + modulatory process model) mutation counts in this cluster. In contrast to model without the modulatory process (Fig. 1D), no systematic deviation is visible

We next applied the inference method to the Liver-HCC cluster discussed above. The fit shows a substantial improvement compared to the NMF additive model, especially in C>G and T>A mutation contexts (Fig. 3A,C). The MSE between predicted and observed mutation counts decreases between two to ten times across all mutation contexts. Aside from removing the structured systematic residues in specific base mutation channels, the modulatory process also decreases the variance of mutation count predictions in base channels where systematic residual patterns were not present.

The inferred modulatory process for the Liver-HCC cluster is highly similar to the mean multiplicative residue of the cluster (Fig. 3A). This indicates an efficient capture of a multiplicatively acting component that was not part of the additive NMF model. The activities of the modulatory process range from 1.15 to 2.5 (Fig. 3B). It is clear that a single modulatory process can in effect remove a large systematic bias with the price of only a modest increase in model complexity which we will discuss shortly. As most samples in this cluster are liver cancers, this modulatory signature may represent some genotoxin-repair process interaction, specific to this cancer type with sample specific intensities.

**Fig. 4:**
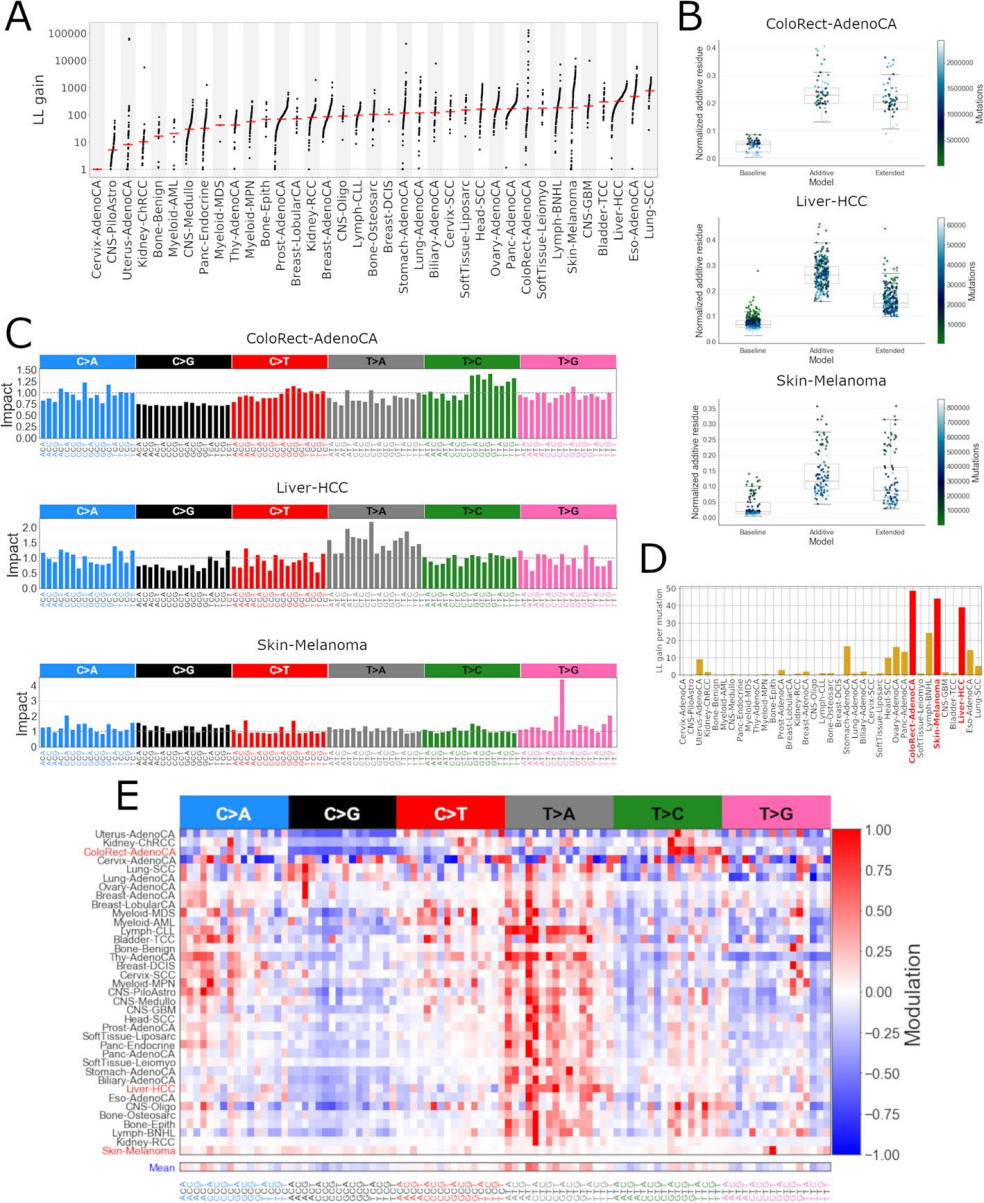
**A** The log-likelihood gain (log_10_ scale) after applying the inferred modulatory processes for each type of cancer. The red line marks the mean. **B** The comparison of additive residues of three models for CRC, melanoma and liver cancers. Baseline refers to the Poisson-resampled model, additive model refers to the NMF model [4] and extended model—the additive model together with the modulatory process. The residues are normalized and colored by total sample-wise mutation burdens. **C** The impact, i.e. the mean of the modulatory process effect in the three selected types of cancer. **D** The total log-likelihood gain, normalized by the total mutation burdens. **E** Hierarchically clustered modulatory signatures of each cancer type. The mean modulatory signature profile across all cancer types is displayed separately at the bottom

Next, we inferred modulatory processes for each cancer type separately. The log-likelihood gain (Fig. 4A) comparing the NMF fits to our extended model show that different cancer types are affected to different extents and there is heterogeneity in terms of modulation between the cancer samples of the same type, i.e. some samples have much higher log-likelihood gain than the rest (e.g. in colorectal adenocarcinoma). Therefore, the inferred modulatory processes are selectively active and there is significant variance in its strength of activity. The median total log-likelihood gain per cancer type (summed over samples) is $\sim 10^4$. The MSE of predicted mutation counts across all PCAWG samples is reduced by a factor of $\sim 2.5$. The inclusion of one modulatory process per cancer type (96 free parameters) and one extra activity per sample is thus comfortably justifiable for majority of cancer types using any reasonable model selection criteria such as the BIC.

We ranked the cancer types according to the total log-likelihood gain, normalized by the number of mutations in the observed mutation catalogues (Fig. 4D). Three cancer types stand out in particular—colorectal adenocarcinoma, skin melanoma and liver hepatocellular carcinoma. There is a clear reduction in the normalized additive residue between NMF additive model and the extended model with the modulatory process (Fig. 4B), especially so in the liver cancer. All three cancer types show different modulatory process impacts (the mean of the modulatory process over the samples, i.e. $\langle 1 + c_k r^j \rangle _k$) (Fig. 4C, Additional file 1: Fig. S4). The inferred modulatory signatures have both some degree of cancer type specificity and common patterns in modulated mutation channel values, furthermore, certain cancers of related organ systems have similar modulatory signatures and are clustered close to each other, e.g. stomach, pancreatic, esophageal, liver and biliary cancers (Fig. 4E).

#### Additive mutational signature activities predict the impact of modulatory processes

We used random forest and linear regression models to assess whether there is a connection between specific mutational signatures from the PCAWG study and the inferred impact of the modulatory process. We trained the models on the sample-wise signature activities of the PCAWG data, to predict the log-likelihood gains. Stratified, five-fold (each fold has the same sample fraction of each cancer type, if possible), cross-validation of the random forest regressor model, repeated 10 times, showed that signature activities are good predictors of the log-likelihood gain, with the mean $R^2$ statistic across the folds being between 0.85 to 0.93 in each run. Interestingly, signature SBS5 is reported to be by far the most important predictor in every fold ($>0.7$ relative importance). However, the linear model performed worse in log-likelihood prediction—the best average $R^2$ statistic was $\sim 0.5$.

## Discussion

We carried out an investigation of mutations left unexplained by the mutational signature analysis done by Alexandrov *et al.* in 2,780 cancer whole-genomes (PCAWG) [4, 15]. While the signature analysis had resulted in an excellent fit in terms of cosine similarity, we were still able to observe non-random structure in the residuals. This structure was evident in samples clustering by tumor type, most notably in a subset of skin melanomas and liver cancers. Potential causes for clustered residuals include biological variability in DNA damage and repair mechanisms active in the tumors, interplay of these mechanisms [16] as well as technical reasons such as the procedure used to extract mutational signatures in PCAWG [4].

To explore the cause of structured residuals, we extended the additive mutational signature model with a multiplicative term capable of correcting both overestimated and underestimated mutation counts. Our modified model, adding only a relatively small number of parameters to the original model, was able to find cancer type specific modulatory processes explaining a large amount of mutations left unexplained by the additive model (Additional file 1: Fig. S3). Particularly in liver, colorectal and skin cancers the improvement in model fit was substantial, perhaps due to the heterogeneity in terms of mutational mechanisms active in these tumor types.

Surprisingly, we found mutational signature SBS5 activity in a tumor to be a strong predictor of inferred modulatory process strength. SBS5 is a clock-like signature with a “featureless” mutation spectrum appearing in most cancer samples across all types [4]. The signature has been associated with aging, tobacco smoking [19] and deficient nucleotide excision repair (NER) [20], but no universally accepted causative mechanism has been established to date. Other studies have connected the exposure of SBS5 with the extent of oxidative DNA damage and NER activity [21] and pointed out cancer-specific transcriptional strand biases of this signature [22]. In some cancers like liver hepatocellular carcinoma, SBS5 contributes most mutations to the mutation types where the systematic deviation is most observed, *i.e.* C>G and T>A. In these mutation types, signature analysis with the additive NMF model results in 24% overestimation in C>G and 32% underestimation of T>A. These discrepancies are quite substantial—in total, there are hundreds of thousands of mutations that are unaccounted for.

Making a definitive disentanglement between DNA damage and repair has proven difficult, even though the DNA repair mechanism is a crucial component, as removing mutations via DNA repair could explain the overestimated mutations. It has been previously shown that the mutagenetic interaction between two defective DNA repair components—polymerase proofreading and mismatch repair (MMR)—could not be summarized as simply an additive product of their separate effects on the genome, but rather that such a combination results in unique mutational profiles [23]. Building upon increasing evidence of non-additive mutational signature interplay, another recent study has also incorporated a similar multiplicative component in mutational signature interaction modelling, and extracted new MMR signatures [24]—however, unlike in that model, our modulatory process is also capable of modelling the reduction, i.e. the removal of the mutation counts, rather than just amplification. Similarly, multiplicative interactions, representing various genomic properties, were modelled in Vohringer *et al.* [25]. Knockouts of *EXO1* gene, coding for a component in MMR and also DNA double-strand break repair (DSBR) pathways [26], have been implicated to cause mutagenetic patterns similar to signature SBS5 [27, 28]. Interestingly, *EXO1* has been also described to have a role in NER pathway [29].

Additionally, we have investigated the possible connection between the log-likelihood gain in our model and PCAWG samples, associated with MMR deficiency, i.e. microsatellite instability (MSI) positive samples. We discovered a positive correlation between the MSI sample mutation burden and the log-likelihood gain ($R^2 = 0.45$). As a follow-up, we have labelled PCAWG samples with >30,000 mutations as high tumour mutation burden (H-TMB) samples and found that they share the same pattern—consistently higher log-likelihood gain within the same cancer type. However, we did not find strong evidence that the per-mutation log-likelihood gain represents a similar connection. For cancer types with at least *n* = 5 MSI samples, namely uterine, stomach and colorectal adenocarcinomas (12/51, 6/75 and 9/60 MSI samples, respectively), only uterine cancers show a significant difference between MSI and non-MSI samples in terms of per-mutation log-likelihood gain (two-sided Kolmogorov-Smirnov test, *p* < 0.0001). While this finding has a potential for further investigation (outside the scope of this study), we believe that this is not yet sufficient evidence to link modulatory processes to the MSI/H-TMB tumour status.

Our proposed multiplicative model is able to summarize the systematic deviations—mutation count overestimation and underestimation—to the point that the remaining variance is reminiscent of Poisson process related noise. One of the aims of our model was that it extends the biologically highly interpretable NMF framework treating the modulatory process as a correction to the standard additive model. In this spirit we did not seek here a *de novo* joint inference of mutational signatures and modulatory processes but took the PCAWG signature set and analysis as the gold standard baseline and built upon that. A joint inference would likely require substantial method development and is clearly beyond the scope of our study. We hope that the modulatory signatures help to concisely represent and summarize this purported DNA damage/repair interaction, which can be quantified at the sample and mutation type levels. We see that the modulatory signatures are often either cancer type-specific or shared amongst cancers of organs with a common role in the human body, e.g. gastrointestinal cancers. While our analysis was performed with the canonical 96 mutation types (*i.e.* triplets), similar analyses can be conducted in larger contexts (*e.g.*, 5-mers) with sufficient data. Nevertheless, we believe that our study with the proposed model gives of an easily inferrable summary of previously undefined DNA repair/damage interactions and undiscovered modulatory signatures, and so identifies an interesting direction where to expand the important signature analysis field.

## Conclusions

The state-of-the-art mutational signature set, derived using additive methods (i.e. NMF) [4], exhibits non-additive properties which are visible through non-random residues between the data and model. An extension to the additive model to include a multiplicative component—a modulatory process—comprehensively addresses these residues. The modulatory processes form cancer specific profiles and may represent sample specific DNA repair propensities. These results are indicative of a promising research direction for the mutational signature analysis field.

## Methods

### Data

We used the mutational signature set, their inferred sample level activities (also known as exposure or mutation attribution) and the corresponding mutation count data from Alexandrov *et al.* study [4] to evaluate the residues using Eqs. 1. The mutation count data was derived from the Pan-Cancer Analysis of Whole Genomes (PCAWG) [15] and consists of 2,780 cancers across 37 cancer types, totalling in 48,276,930 mutations. The residues quantify any discrepancies between observed versus model predicted mutational counts in the single-base substitution (SBS) somatic mutation class. The mutational signature set, extracted using NMF-based *SigProfiler* method in the original study, forms the basis of the contemporary mutational signature database in the Catalogue Of Somatic Mutations In Cancer (COSMIC) [30], and is used as the primary mutational signature set for this analysis. Qualitatively similar results are also evident using the alternative mutational signature set and mutation-sample attributions from the Bayesian-NMF method, *SignatureAnalyzer* [4]. All these data are obtained from the publicly accessible Synapse repository (accession ID: syn11726601). Additionally, a list of PCAWG samples with the microsatellite instability (MSI) positive label have been obtained from *SignatureAnalyzer* study source code repository at https://github.com/getzlab/SignatureAnalyzer. To avoid infinite values in division, i.e. multiplicative residue calculation and also logarithmic transformation, a pseudo-count of 1 is added to all mutation counts of every mutation type.

### Hierarchical clustering

Unsupervised hierarchical clustering was performed using *scipy* [31] interface (*scipy.cluster.hierarchy*). Multiplicative residue correlations are clustered using complete linkage (farthest-neighbor clustering) using a pre-defined threshold, which in this analysis is 0.75—this number is chosen arbitrarily to balance the number of clusters and their sizes (e.g. to avoid one-sample clusters). In the triangularly split heatmap Fig.  1, the upper triangle represents the multiplicative residue correlations, and the lower—additive residue correlations (the sample order is the same as in the upper triangle, i.e. for both residues, the sample order is defined by only the multiplicative residue correlations). We calculate the entropy as $S = -\sum _{i}p_i \log (p_i)$, where $p_i$ is the *i*-th cancer type probability in the cluster (calculated from the frequencies of the cancer type labels) and we only consider the clusters that have at least 10 samples.

### Additive model

Having 6 base mutation types (only the pyrimidine-base of the mutated base pair is considered)—C>A, C>G, C>T, T>A, T>C, T>G—mutations are considered in a trinucleotide context (one base from both 5’ and 3’ sequence ends), totalling in 96 mutation type mutational signature. The signatures are normalized so that they sum up to 1, i.e. a mutational signature is a probability distribution over 96 features. The standard mutational signature model assumes a linear model, i.e. the predicted mutational catalogue (spectra) is an additive product of weighted mutational signatures. Given the number of samples $N_S$, number of channels/features $N_C$ and number of mutational signatures $N_{\textrm{Sig}}$, it can be mathematically expressed as follows:

3 $$\begin{aligned} {\widetilde{X}}^k_j = \sum _{i = 1}^{N_{\textrm{Sig}}} a_i^k\mu _j^i \end{aligned}$$

Here ${\widetilde{X}}^k_j, k = 1,\dots ,N_S, j = 1,\dots ,N_C$ is the model predicted mutation count for *j*-th channel of sample *k*, $N_{\textrm{Sig}}$ is the number of mutational signatures, $\mu _j^i$ is the mutation probability in *j*-th channel of *i*-th mutational signature and $a_i^k$ is the activity of *i*-th mutational signature in *k*-th sample.

### Data simulation

The simulated mutation catalogues are created by taking 10 random mutational signatures $\mu$ from COSMIC (to simulate a biologically plausible cancer type), of which random 4 are selected to be active in a sample, simulating their non-negative real-valued activities *a* and then affected by a simulated modulatory process *r* with randomly drawn activities *c*. The activities *a* are independently drawn from a log-normal distribution with scale 1.5 and mean 0, and the initial mutation counts are given by the dot product of *a* and $\mu$. Under the Poisson model, multiplying the resulting mutation intensities with the chosen modulatory process *r* and the respective activities *c* gives the synthetic mutation spectra, which are then used as Poisson rates to simulate the “observed” data. The linear combination of the signatures $\mu$ (kept fixed) and the activities *a* (that are inferred *de novo* with the log-likelihood maximization algorithm) give predicted mutational catalogues, representing the additive simulated model fit that does not take into account the modulatory process.

In each simulated data instance the simulated modulatory process *r* affects the mutation channels in two out of 6 SBS base classes so that the mutation channels in one have only positive values and in the other—only negative. The value that determines the modulation in each mutation channel of the selected base type (e.g. C>G) is drawn from ${\mathcal {U}}(0, 1)$, additionally, noise drawn from ${\mathcal {N}}(0, 0.05)$ is added for all 96 features. The strength of modulation effect is dependent on the modulatory process activity vector *c*, which is randomly drawn from ${\mathcal {U}}(0, 1)$. Both simulated *r* and *c* are gauged, meaning that *r* is divided and *c* is multiplied with the highest absolute value of *r* (furthermore, the modulatory process cannot act negatively, i.e. $r_j$ and $c^k$ have to meet the condition $1 + c^k r_j \ge 0$). The gauge plays a role similar to the normalisation of each additive signature $\mu _i$ to sum up to 1 and removes a degenerate direction from the inference.

### Modulatory process inference by maximizing the log-likelihood

The modulatory process and its activities in samples are inferred using maximum likelihood method by gradient ascent. Given the number of samples $N_S$, number of mutation types (channels) $N_C$, number of mutational signatures $N_{\textrm{Sig}}$, the additive model fit ${\widetilde{X}}^k_j = \sum _{i = 1}^{N_{\textrm{Sig}}} a_i^k\mu _j^i$ and the active modulatory process $p^k_j = 1 + c^k r_j$, the likelihood for the data $X^k_j$ under Poisson model (Eq. 1) is given by the function

4 $$\begin{aligned} p(X | \mu , a, r, c) = \displaystyle \prod _{k = 1}^{N_{S}} \prod _{j = 1}^{N_{C}} \frac{e^{-p^k_j{\widetilde{X}}^k_j}(p^k_j{\widetilde{X}}^k_j)^{X^k_j}}{X^k_j!} \end{aligned}$$

The log-likelihood of the above is given by

5 $$\begin{aligned} \log (p(X | \mu , a, r, c)) = \sum _{k = 1}^{N_{S}} \sum _{j = 1}^{N_{C}} - p^k_j {\widetilde{X}}^k_j + X^k_j \log (p^k_j {\widetilde{X}}^k_j) - \log (X^k_j!) \end{aligned}$$

Since the factorial of $X^k_j$ is simply an additive constant and not relevant to the log-likelihood score, it is removed for subsequent calculations. The derivatives with respect to *a*, *r* and *c* ($\mu$ are treated as given in the inference algorithm) are:

6 $$\begin{aligned}{} & {} \frac{\delta \log (p(X | \mu , a, r, c))}{\delta a^k_i} = \sum _{j = 1}^{N_{C}} \frac{X^k_j \mu ^i_j}{{\widetilde{X}}^k_j} - p^k_j \mu ^i_j \end{aligned}$$

7 $$\begin{aligned}{} & {} \frac{\delta \log (p(X | \mu , a, r, c))}{\delta r_j} = \sum _{k = 1}^{N_{S}} \frac{X^k_j c^k}{p^k_j} - c^k {\widetilde{X}}^k_j \end{aligned}$$

8 $$\begin{aligned}{} & {} \frac{\delta \log (p(X | \mu , a, r, c))}{\delta c^k} = \sum _{j = 1}^{N_{C}} \frac{X^k_j r_j}{p^k_j} - r_j {\widetilde{X}}^k_j \end{aligned}$$

The optimization is done with *scipy.minimize* interface using truncated Newton (TNC) algorithm, passing the log-likelihood score and parameter-specific derivative functions described above. The TNC algorithm restarts with randomly initialised parameter values until a successful convergence is attained or the inference loop exceeds 100 attempts. For the simulated data benchmark, the activity and modulatory process inference is iterative, i.e. repeated several times (three by default) inferring one after the other.

After every iteration of the optimization algorithm, both *r* and *c* are gauged (see “Data simulation” section) to ensure favorable direction of the gradient.

## Supplementary Information

**Additional file 1.** Supplementary Figures.

## Data Availability

Python 3.7.6 code, related IPython notebooks and the initial data for the analysis described in this manuscript can be found at https://www.github.com/dovydask/mp_sig.
