# Supplementary material for: Identification of multiplicatively acting modulatory mutational signatures in cancer
Source: BMC Bioinformatics. 2022 Dec 6;23:522. doi: 10.1186/s12859-022-05060-8 (PMC9724449; doi:10.1186/s12859-022-05060-8)
Supplement: Supplementary file 1 — Additional file 1. Supplementary Figures. [file 12859_2022_5060_MOESM1_ESM.pdf]

Supplementary Figures

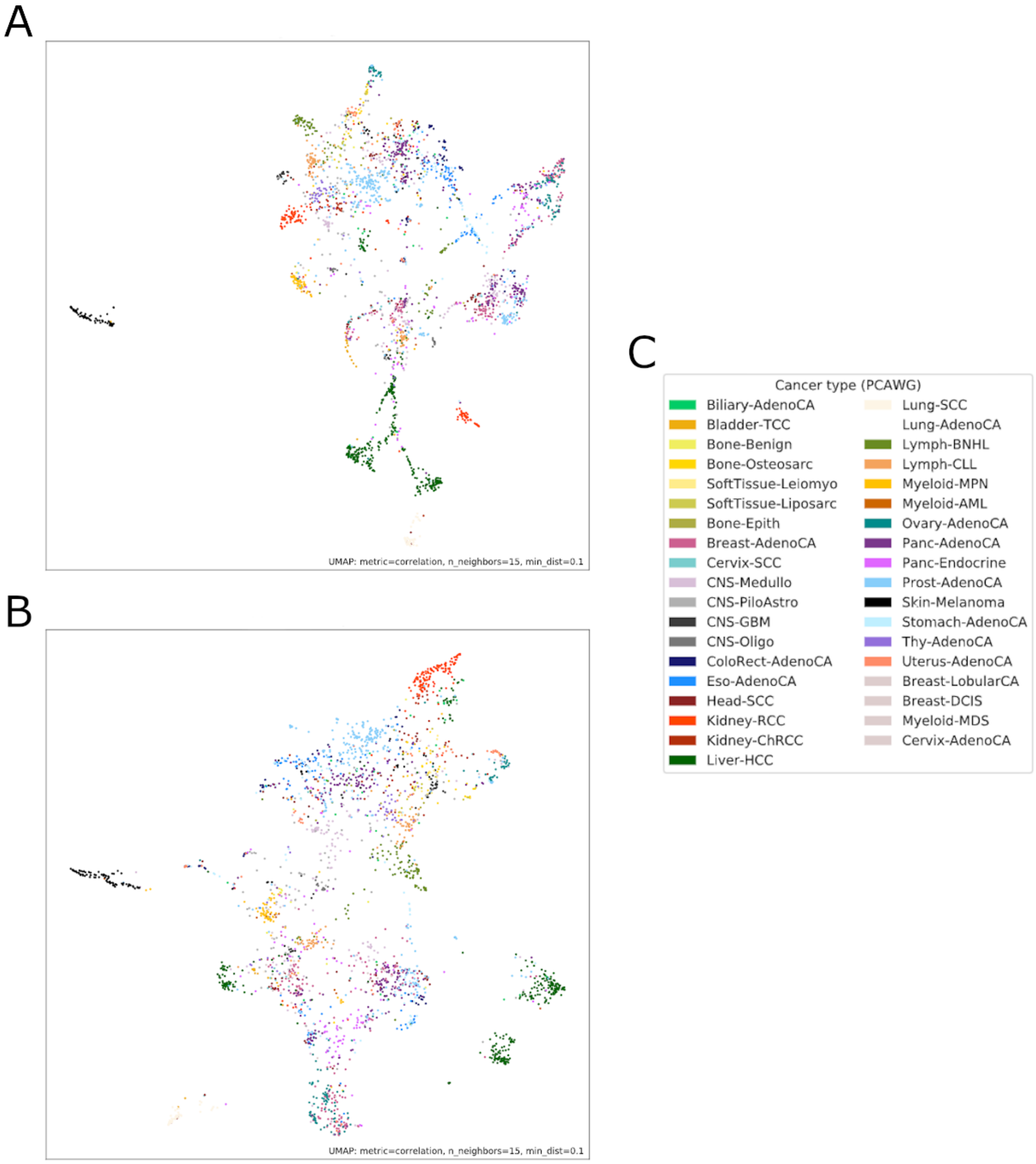

Supplementary Figure 1: **A** Two-dimensional UMAP of additive residues of the observed versus additive model-predicted PCAWG mutation count. **B** Two-dimensional UMAP of multiplicative residues of the observed versus additive model-predicted PCAWG mutation counts. **C** Color legend for PCAWG cancer types.

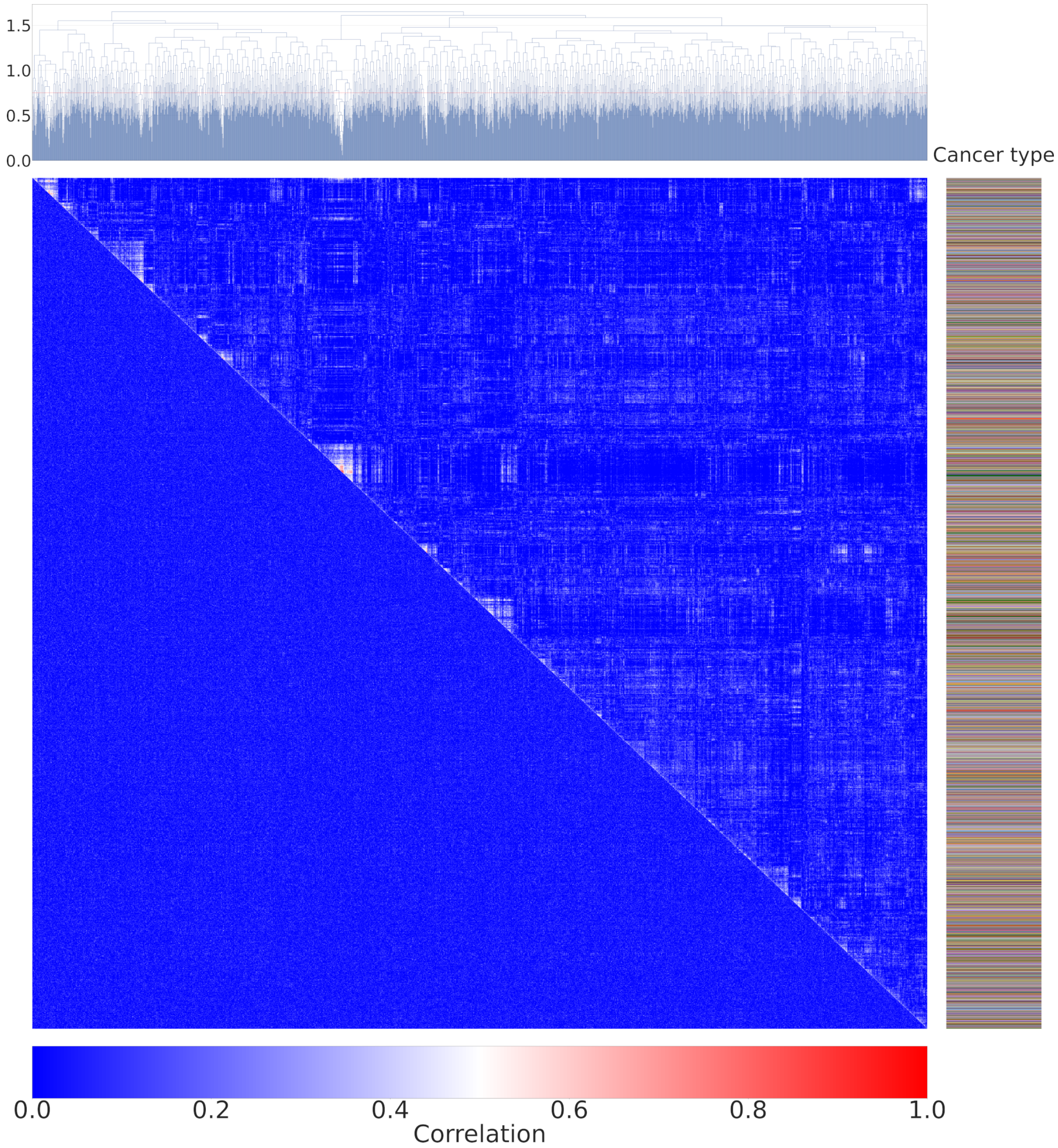

Supplementary Figure 2: A heatmap of the residue correlations of observed counts versus Poisson-resampled mutation counts serving as a 'perfect' control model. The residues reflect Poisson noise and have no structure. Upper triangle represents multiplicative residues and the lower – additive (here the samples are ordered in the same order as in the upper triangle).

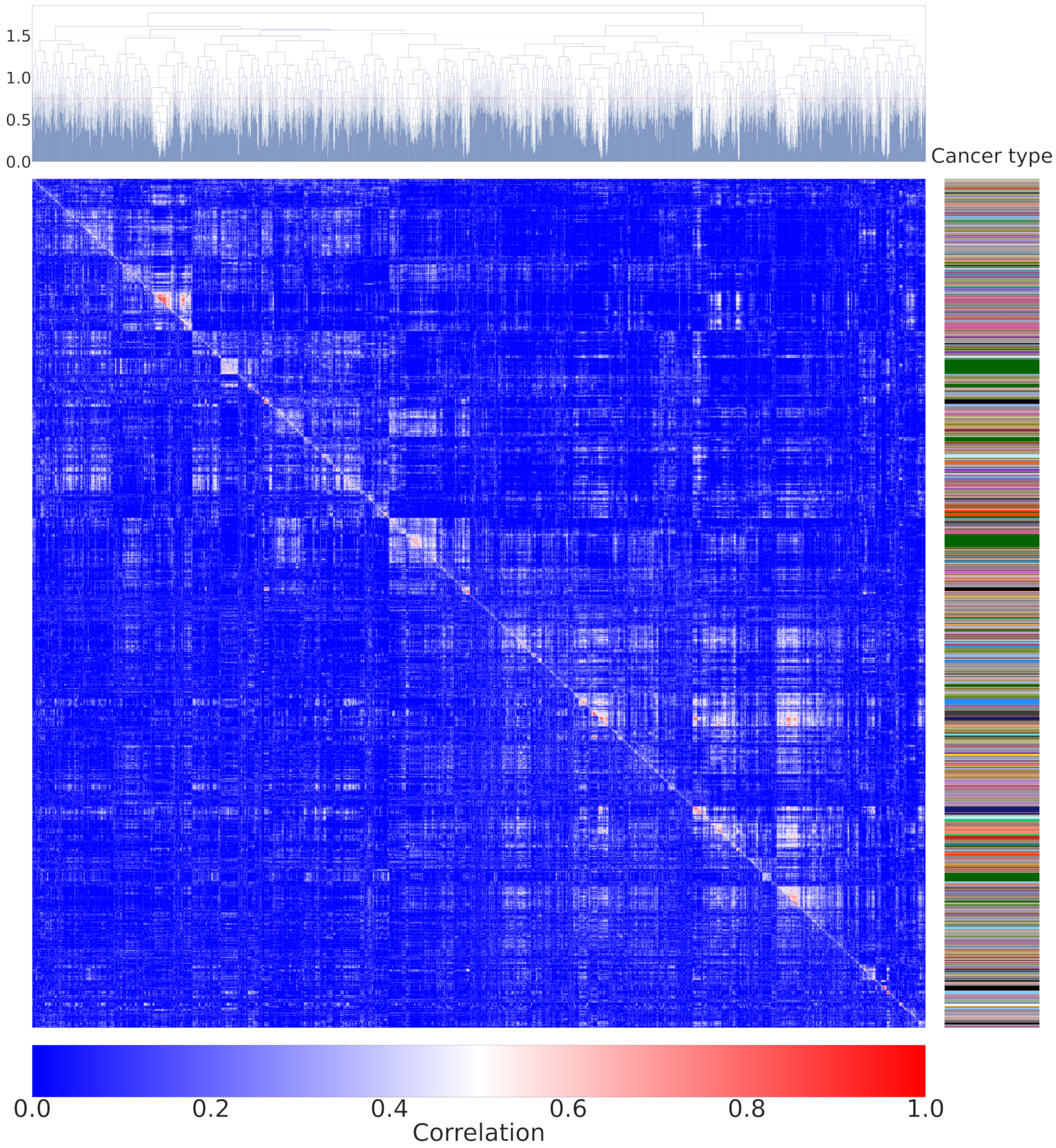

Supplementary Figure 3: The heatmap of residue correlations of PCAWG-data with respect to the extend model (upper – multiplicative, lower – additive, samples ordered by the former). The structure shown in Figure 1 has almost fully vanished indicating that no systematic bias in the fit is left.

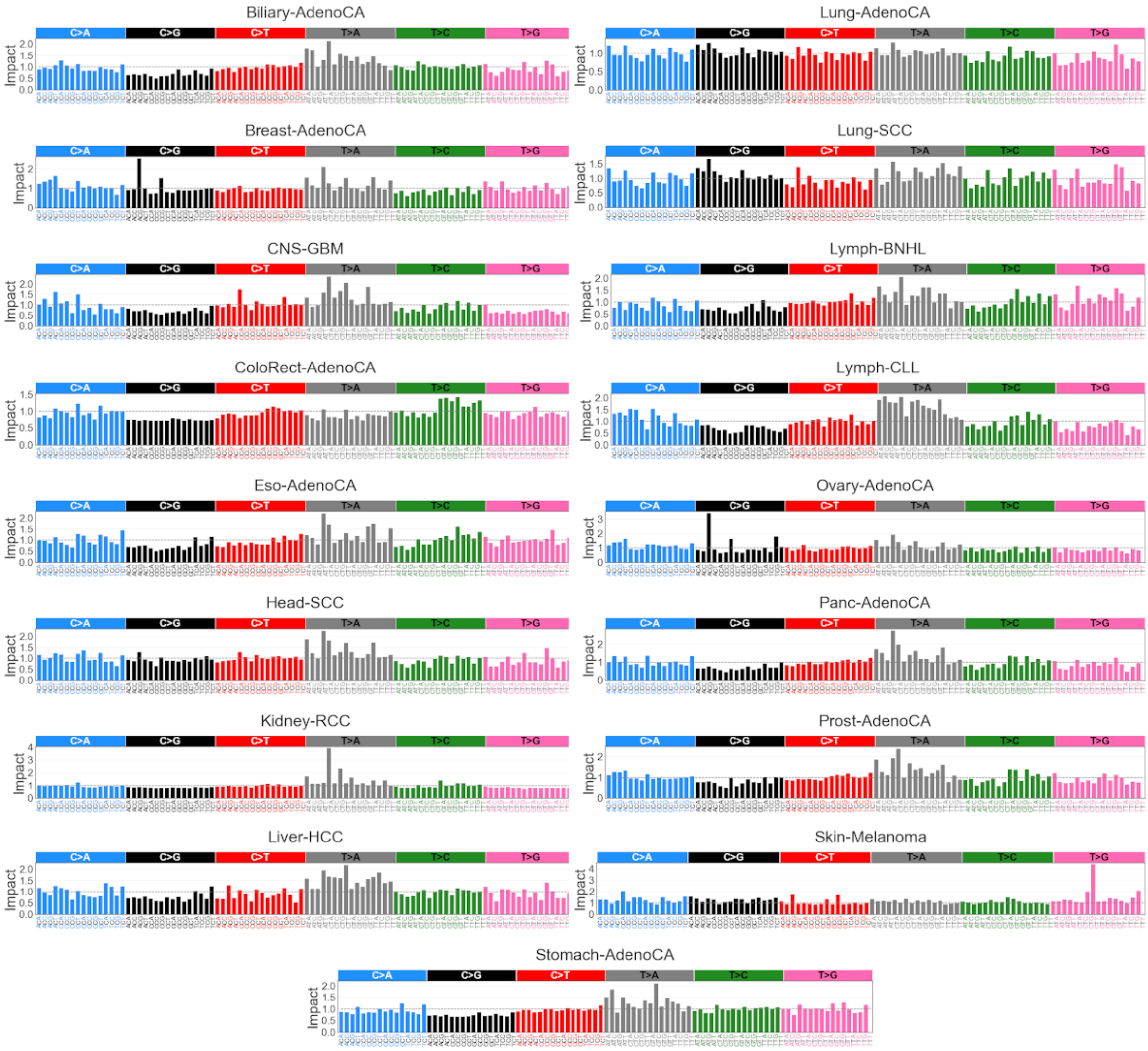

Supplementary Figure 4: Impacts (mean effect of the inferred modulatory process) of 17 cancers for which the total combined log-likelihood gain after affecting them with their respective inferred modulatory processes exceeds 10,000 log-likelihood score units (see Figure 4A). While Uterus-AdenoCA satisfies this condition, it is excluded as only 2 samples contribute to almost all log-likelihood gain for this cancer type.

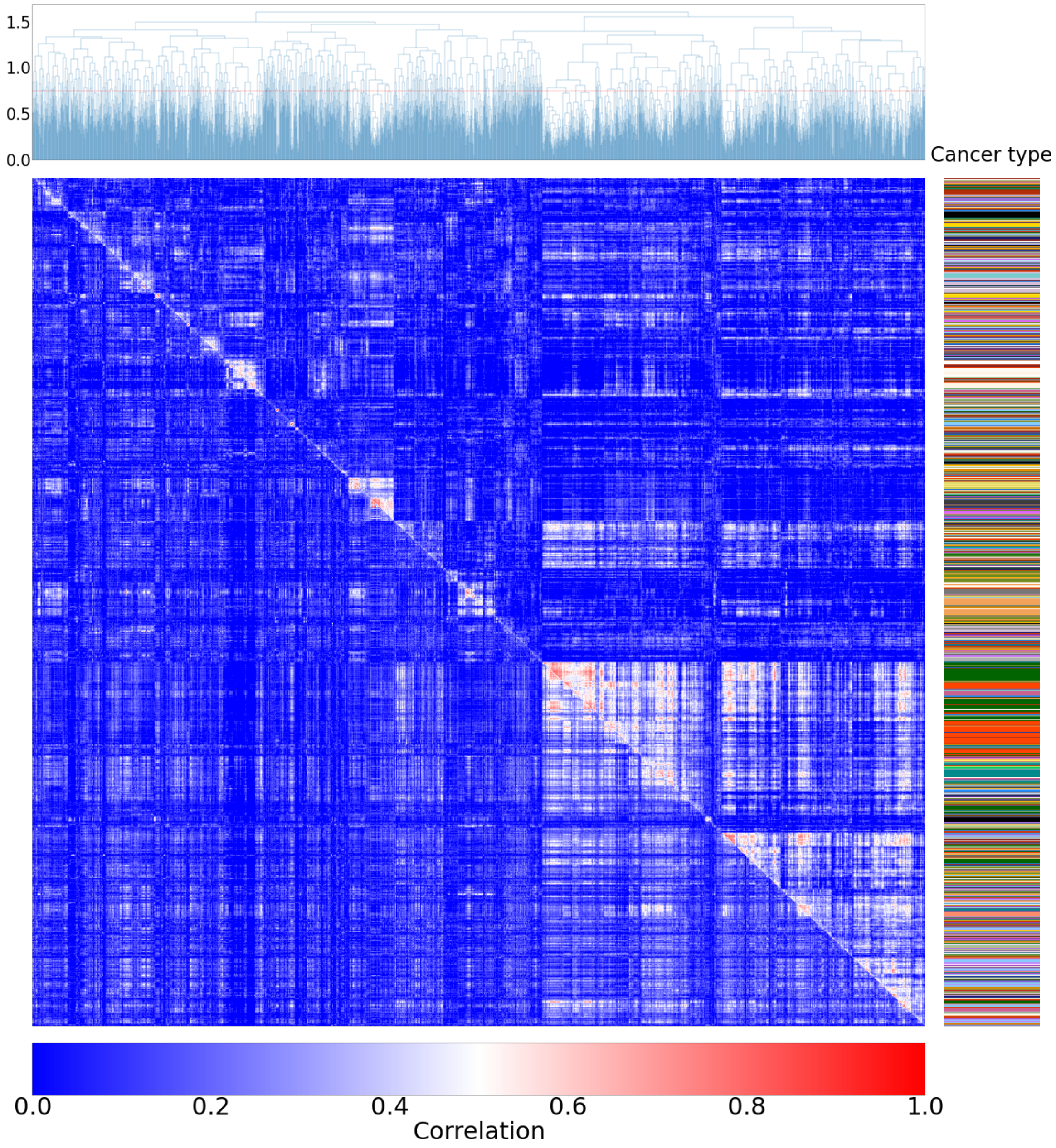

Supplementary Figure 5: A heatmap of the residue correlations of observed versus *SignatureAnalyzer* model fit observed mutation count residues. Upper triangle represents multiplicative residues and the lower – additive (here the samples are ordered in the same order as in the upper triangle).
